# Marmoset glutathione transferases with ketosteroid isomerase activity

**DOI:** 10.1016/j.bbrep.2021.101078

**Published:** 2021-07-12

**Authors:** Aram Ismail, Julia Sawmi, Bengt Mannervik

**Affiliations:** Department of Biochemistry and Biophysics, Arrhenius Laboratories, Stockholm University, SE-10691, Stockholm. Sweden

**Keywords:** 5-Androsten-3,17-dione, 5-Pregnen-3,20-dione, CjaGST A3-3, CjaGST A1-1, Alpha glutathione transferase, Steroid hormone synthesis, Glutathione transferase, (GST), Glutathione, (GSH), 5-androsten-3,17-dione, (Δ^5^-AD), 5-pregnen-3,20-dione, (Δ^5^-PD), 4-androsten-3,17-dione, (Δ^4^-AD), 1-chloro-2,4-dinitrobenzene, (CDNB), phenethyl isothiocyanate, (PEITC), allyl isothiocyanate, (AITC), SDS-PAGE, (sodium dodecyl sulfate-polyacrylamide gel electrophoresis)

## Abstract

The common marmoset *Callithrix jacchus* encodes two glutathione transferase (GST) enzymes with ketosteroid double-bond isomerase activity. The most active enzyme is CjaGST A3-3 showing a specific activity with 5-androsten-3,17-dione (Δ^5^-AD) of 62.1 ± 1.8 μmol min^-1^ mg^-1^, and a k_cat_ value of 261 ± 49 s^-1^. The second ketosteroid isomerase CjaGST A1-1 has a 30-fold lower specific activity with Δ^5^-AD and a 37-fold lower k_cat_ value. Thus, the marmoset CjaGST A3-3 would be the main contributor to the biosynthesis of the steroid hormones testosterone and progesterone, like the human ortholog HsaGST A3-3. Two residues differ in the H-site of the 91.4% sequence identical CjaGST A1-1 and CjaGST A3-3, and modeling of the structures suggests that the bulky phenyl ring of Phe111 in CjaGST A1-1 causes steric hindrance in the binding of the steroid substrate. Tributyltin acetate (IC_50_=0.16 ± 0.004 μM) and ethacrynic acid (IC_50_=3.3 ± 0.2 μM) were found to be potent inhibitors of CjaGST A3-3, as previously demonstrated with the human and equine orthologs.

## Highlights

• Marmoset glutathione transferase A3-3 displays potent ketosteroid isomerase activity.
• Marmoset glutathione transferase A1-1 shows weak ketosteroid isomerase activity.
• A model of marmoset A1-1 suggests active-site Phe to interfere with steroid binding.
• Common marmoset monkey – a possible model animal for steroid biosynthesis research.

## 1 Introduction

**Fig. 1:**
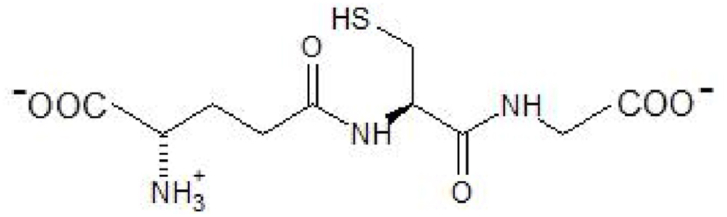
Structure of glutathione, L-γ-glutamyl-L-cysteinyl-glycine.

Glutathione (GSH, Fig. 1) is a tripeptide composed of cysteine, glycine, and glutamic acid, which is abundantly occurring in the cells of higher organisms.

The thiol group of the cysteinyl residue serves as an antioxidant and a scavenger of electrophiles in the cellular defense against toxic agents. The detoxication reactions are catalyzed by enzymes of which glutathione transferases (GSTs) play a prominent role. GSTs occur in three structurally distinct families of cytosolic, mitochondrial and membrane-associated proteins. The mammalian cytosolic GSTs occur in seven different classes called Alpha, Sigma, Mu, Omega, Pi, Zeta, and Theta. Five different genes in the human genome encode Alpha class GSTs, which form homodimeric or heterodimeric structures.

**Fig. 2:**
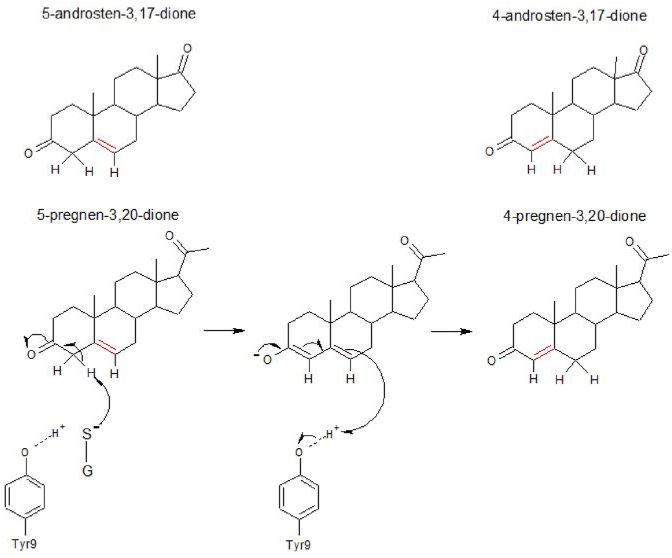
Double-bond isomerization catalyzed by GSTs with ketosteroid isomerase activity. The reaction with 5-adrosten-3,17-dione is analogous to the 5-pregnen-3,20-dione isomerization shown. The thiolate of glutathione serves as a base removing a C4 proton, and the steroid is then reprotonated via the active-site Tyr9.

Remarkably, human HsaGST A3-3 is abundant in steroidogenic tissues and displays high ketosteroid double-bond isomerase activity with 5-androsten-3,17-dione (Δ^5^-AD) and 5-pregnen-3,20-dione (Δ^5^-PD) (Fig. 2), precursors of the sex hormones testosterone and progesterone [1].

Downregulation of GST A3-3 expression by RNA interference or inhibition of the enzyme activity suppresses steroid hormone production, suggesting that the enzyme could be a target for pharmacological interventions in conditions characterized by overproduction of hormones [2]. Even though the mammalian steroid isomerase activity was first noted in rodents [3], neither mice nor rats have an enzyme with an efficiency approaching that of human GST A3-3. So far, the only other known mammalian enzymes with conspicuously high ketosteroid isomerase activity are equine GST A3-3 [4] and porcine GST A2-2 [5]. The large animals featuring these enzymes are not ideal for tests of novel drugs, and a primate model would appear more suitable. The common marmoset monkey (*Callithrix jacchus*) is used as an experimental animal, and its genome is available [6]. In preliminary experiments we expressed CjaGST A1-1, the Alpha class GST with the highest overall sequence similarity with human GST A3-3, and demonstrated that it catalyzes the steroid isomerization although with modest efficiency [7]. We now report that the proper ortholog in the marmoset is CjaGST A3-3, which is a more prominent ketosteroid isomerase.

## 2 Materials and methods

### 2.1 Extracting plasmid DNA from GF/C filter

DNA encoding CjaGST A1-1 and A3-3 was synthesized by ATUM (Newark, CA, USA) and delivered on a GF/C filter. The filter was placed in a 0.6 mL tube punctured at the bottom with the help of a syringe and 100 μL of 10 mM Tris-HCl pH 7.5 was added. After 2 min the 0.6 mL tube was placed in a 1.5 mL tube and centrifuged for 1 min at 10 000×*g*, yielding 90 μL buffer containing the extracted DNA at a concentration of 20 ng/μL.

### 2.2 Transformation by heat shock

*Escherichia coli* BL21 (DE3) cells from a -80 °C freezer were placed on ice for 50 min after which 2 μL (40 ng) of the extracted DNA was mixed with 20 μL of the *E. coli* BL21 cells. The mixture was then kept on ice for 30 min before the cells were heat shocked in a 42 °C water bath for 45 s and then put on ice for 2 min. LB broth (250 μL) was added and the bacteria were grown at 37 °C in a shaking incubator for 45 min. The transformed cells were plated on agar with ampicillin (50 μg/mL) and incubated overnight at 37 °C.

### 2.3 Expression and purification of the recombinant GSTs

An overnight culture was prepared by resuspending a colony of transformed bacteria in 50 mL LB medium containing 50 μg/mL ampicillin. The bacterial culture was incubated overnight at 37 °C with 200 rpm shaking. A flask containing 500 mL expression medium (2-TY, 8 g bacto-tryptone, 5 g yeast extract, and 2.5 g NaCl) containing 25 mg ampicillin was inoculated with 5 mL bacteria overnight culture and grown at 37 °C, 200 rpm. When OD_600_ had reached 0.4 expression of enzyme was induced with 0.2 mM isopropyl β-d-1-thiogalactopyranoside and the bacteria were incubated an additional 3 h at 37 °C. The culture was then centrifuged for 7 min at 7000×*g*. The bacterial pellet was mixed with 10 mL lysis buffer (20 mM sodium phosphate, 20 mM imidazole, 0.5 M NaCl pH 7.4, 0.2 mg/mL lysozyme, and one complete mini tablet) and was then incubated for 1h. After the incubation, the cells were disrupted by sonication and the lysate was centrifuged for 30 min at 27 000×*g* in a JA-25.50 rotor.

The synthesized DNA encoded a hexahistidine tag at the N-terminus of the GSTs in order to facilitate their purification by immobilized metal affinity chromatography (IMAC) [8] using a Ni-IMAC column (GE Healthcare). The column was equilibrated with the binding buffer (20 mM sodium phosphate, 20 mM imidazole, 0.5 M NaCl, pH 7.4) before the lysate was applied to the column. The column was washed continuously with the binding buffer until all impurities were removed. An elution buffer (20 mM sodium phosphate, 500 mM NaCl, 250 mM imidazole pH 7.4) was used to release the protein from the column, and the fractions showing the highest absorbance at 280 nm were collected. The pooled fractions were dialyzed two times against 10 mM Tris-HCl, pH 7.8, 1 mM EDTA, 0.2 mM tris(2-carboxyethyl)phosphine. The protein concentration of the final preparation was determined spectrophotometrically at the wavelength 280 nm. An SDS-PAGE (sodium dodecyl sulfate-polyacrylamide gel electrophoresis) followed by staining with Coomassie Brilliant Blue G250 was made to verify the purity of the dialyzed enzyme.

### 2.4 Specific activity determinations

The purified GSTs were characterized by measuring the catalytical activities with alternative substrates. The conditions are given in Table 1 as are the wavelengths used for the spectrophotometric assays.

**Table 1:** Reaction conditions for the tested substrates: 5-androsten-3,17-dione (Δ^5^-AD), 5-pregnen-3,20-dione (Δ^5^-PD), 1-chloro-2,4-dinitrobenzene (CDNB), phenethyl isothiocyanate (PEITC), allyl isothiocyanate (AITC). In assays with Δ^5^-AD and Δ^5^-PD 0.1% (w/v) bovine serum albumin was present. Initial rates were determined at 30 °C and corrected for the nonenzymatic background reaction. The final concentration of enzyme differed for the diverse substrates; the range of CjaGSTA1-1 concentrations was 0.15–5.1 μg/ml and the range for CjaGST A3-3 was 0.21–10.6 μg/ml.

Substrate	[Substrate] (mM)	[GSH] (mM)	Buffer, pH	Wavelength (nm)
Δ^5^-AD	0.1	1.0	0.25 mM sodium phosphate, 1 mM EDTA, pH 8	248 ε = 16.3 mM^-1^ cm^-1^
Δ^5^-PD	0.01	1.0	0.25 mM sodium phosphate, 1 mM EDTA, pH 8	248 ε = 17.0 mM^-1^ cm^-1^
CDNB	1.0	1.0	0.1 M sodium phosphate, 1 mM EDTA, pH 6.5	340 ε = 9.60 mM^-1^ cm^-1^
PEITC	0.4	1.0	0.1 M sodium phosphate, 1 mM EDTA, pH 6.5	274 ε = 8.89 mM^-1^ cm^-1^
AITC	0.4	1.0	0.1 M sodium phosphate, 1 mM EDTA, pH 6.5	274 ε = 7.45 mM^-1^ cm^-1^

### 2.5 Modeling of protein structures

Molecular graphics and analyses were performed with 10.13039/100008069UCSF Chimera, developed by the Resource for Biocomputing, Visualization, and Informatics at the 10.13039/100008069University of California, San Francisco, CA, 10.13039/100011408USA with support from NIH P41-GM103311 [9]. Chimera interfaced to Modeller [10] was used for homology modeling of the marmoset GST structures based on PDB ID: 2VCV.

## 3 Results

### 3.1 Purification of CjaGST A1-1 and CjaGST A3-3

The enzymes were obtained in high yield by heterologous expression in *E. coli* and purified by Ni-IMAC. The purified proteins showed a single band at approximately 25 kDa when subjected to SDS–PAGE. In the crude lysate the same band was the dominating component. The protein concentration and the total amount of enzyme were 10.1 mg/mL and 28 mg for CjaGST A1-1 and 10.6 mg/mL and 25 mg for CjaGST A3-3.

### 3.2 Comparison of primary structures

**Fig. 3:**
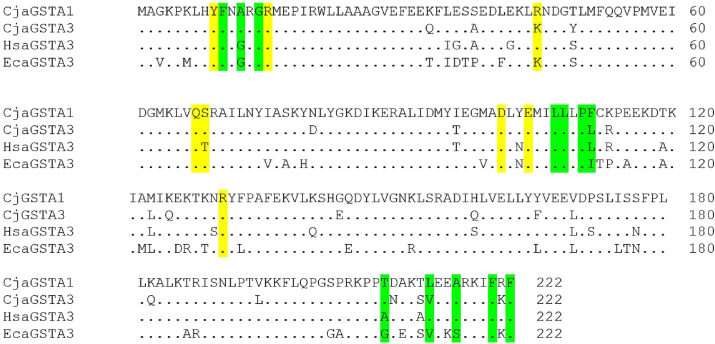
Alignment of the amino acid sequences of the Alpha class enzymes CjaGST A1-1, CjaGST A3-3, HsaGST A3-3, and EcaGST A3-3. G-site residues are indicated in yellow, while H-site residues are indicated in green. (For interpretation of the references to color in this figure legend, the reader is referred to the Web version of this article.)

The genes of the marmoset enzymes studied encode primary structures of 222 amino acid residues, and the sequences are 91.4% identical (Table 2). For comparison the primary structures of the human and equine enzymes HsaGST A3-3 and EcaGST A3-3, respectively, are shown (Fig. 3). Overall, the CjaGST A1-1 sequence showed slightly higher identity with both the HsaGST A3-3 and EcaGST A3-3 sequences than did the CjaGST A3-3 sequence (Table 2). All soluble mammalian GSTs are dimers and the marmoset enzymes are accordingly designated CjaGST A1-1 and CjaGST A3-3 to indicate their composition of two identical subunits, A1 and A3, respectively [11].

**Table 2:** Sequence identities (%) among related alpha class GSTs.

	CjaGSTA1	CjaGSTA3	HsaGSTA3	EcaGSTA3
CjaGSTA1	100	91.4	90.5	80.2
CjaGSTA3	91.4	100	88.3	79.3
HsaGSTA3	90.5	88.3	100	80.6
EcaGSTA3	80.2	79.3	80.6	100

The active site of GSTs is formed by two parts called the G-site and the H-site [12,13], which are responsible for binding glutathione and the second substrate, respectively. The G-site comprises Tyr9, Arg15, Lys/Arg45, Gln67, Thr/Ser68, Asp101 (contributed by the neighboring subunit), Glu104, Arg131 (from the neighboring subunit) and is conserved in all four GSTs compared. The H-site, which forms a largely hydrophobic cavity, in Alpha class enzymes includes residues 10, 12, 14, 15 (side-chain methylene group), 107, 108, 110, 111, 208, 213, 216, 220, and 222 (Table 3). These H-site residues are major contributors to the substrate selectivity of a given GST. The most noteworthy difference among the enzymes in Table 2 is Phe in position 111 of CjaGST A1-1, where the other proteins have Leu/Ile.

**Table 3:** Comparison of H-site residues in CjaGST A1-1, CjaGST A3-3, HsaGST A3-3, and EcaGST A3-3. The human and equine enzymes are the most efficient ketosteroid isomerases and have similar specific activities, suggesting that the structural differences in positions 111, 213, and 216 are without consequence. The H-site residues of CjaGST A3-3 match either one or the other of the most efficient orthologs with the exception of Thr208, the latter residue possibly responsible for the 3-fold lower specific activity. The H-site residues of the two marmoset enzymes differ in positions 111 and 213, of which the voluminous Phe111 in CjaGST A1-1 may interfere with steroid binding and explain the considerably lower ketosteroid isomerase activity.

Residue	CjaGSTA1	CjaGSTA3	HsaGSTA3	EcaGSTA3
10	F	F	F	F
12	A	A	G	G
14	G	G	G	G
15	R	R	R	R
107	L	L	L	L
108	L	L	L	L
110	P	P	P	P
111	F	L	L	I
208	T	T	A	G
213	L	V	L	V
216	A	A	A	S
220	F	F	F	F
222	F	F	F	F

Crystal structures have been determined for HsaGST A3-3 [14] and EcaGST A3-3 [15], both of which show the highest ketosteroid isomerase activity of all known mammalian enzymes. The structures of the homologous enzymes are very similar and the structures include a ternary complex of the human enzyme with both glutathione and the steroid Δ^4^-AD as ligands in the G-site and the H-site, respectively.

**Fig. 4:**
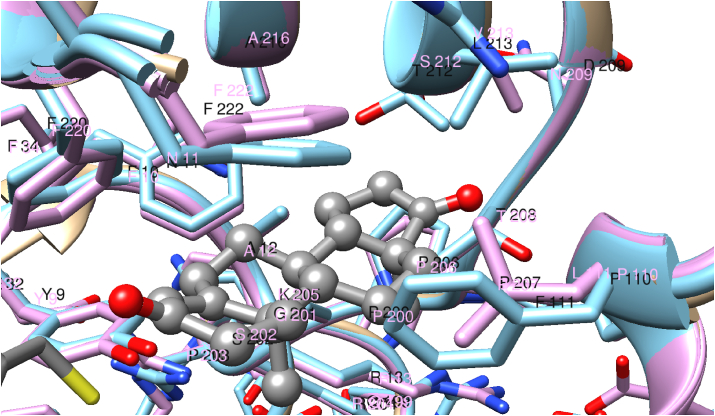
Active-site models of CjaGST A1-1 (light blue) and CjaGST A3-3 (pink) based on the crystal structure of HsaGST A3-3 in complex with 4-androsten-3,17-dione. The steroid (colored by element) is rendered in ball and stick. The functional thiol of glutathione (yellow) and Tyr9 are shown in the lower left corner, adjacent to the steroid. (For interpretation of the references to color in this figure legend, the reader is referred to the Web version of this article.)

Owing to the high sequence similarities the structures of the marmoset GSTs can be derived by homology modeling. Both CjaGST A1-1 and CjaGST A3-3 were very similar in structure to the other GSTs. Fig. 4 shows an overlay of the two modeled marmoset proteins and a closeup of Δ^4^-AD in the H-site obtained from the ternary complex of HsaGST A3-3. CjaGST A3-3 can accommodate the steroid in a similar manner as HsaGST A3-3, but CjaGST A1-1 leaves less space for the steroid primarily owing to the bulky sidechain of Phe111, where the other enzymes display Leu/Ile111. Δ^4^-AD is actually the product of the isomerase reaction, but the chemical structure of the isomeric substrate Δ^5^-AD differs only in the position of a double bond and the altered puckering of the A and B rings of the steroid.

### 3.3 Substrate specificities of CjaGST A1-1 and CjaGST A3-3

Both CjaGST A1-1 and CjaGST A3-3 demonstrated unequivocal ketosteroid isomerase activity, but the latter enzyme was 20 to 30-fold more active than the homologous enzyme (Table 4). Noteworthy enzyme activity was dependent on the presence of glutathione in the assay system. The specific activity of 62 μmol min^-1^ mg^-1^ for CjaGST A3-3 with Δ^5^-AD as a substrate is the most prominent among its activities with the substrates tested. Thus, the observed isomerase activities are in agreement with the modeling of the marmoset enzymes (Fig. 4).

**Table 4:** Specific activities CjaGST A3-3 and CjaGST A1-1 with alternative substrates. The measurements were carried out in triplicate under conditions described in Table 1.

Specific activity (μmol min^-1^ mg^-1^)		
Substrate	CjaGST A3-3	CjaGST A1-1
Δ^5^-AD	62.1 ± 1.77	1.96 ± 0.08
Δ^5^-PD	4.57 ± 0.27	0.22 ± 0.02
CDNB	13.1 ± 0.24	26.1 ± 0.59
PEITC	4.92 ± 0.28	8.80 ± 1.95
AITC	3.15 ± 0.11	6.01 ± 0.61

The specific activity with Δ^5^-PD is only 4.57 μmol min^-1^ mg^-1^, but the comparison is not unbiased, since the substrate concentration used is 10-fold lower because of the limited solubility of Δ^5^-PD in the assay medium. The CDNB activity is 5-fold lower than the Δ^5^-AD value, whereas both isothiocyanate substrates give approximately 10 to 20-fold lower specific activities in comparison with Δ^5^-AD.

In the case of CjaGST A1-1 the highest specific activity 26 μmol min^-1^ mg^-1^ was found with CDNB, 13-fold higher than the activity with Δ^5^-AD. The second highest specific activity of 8.8 μmol min^-1^ mg^-1^ was obtained with PEITC followed by 6.0 μmol min^-1^ mg^-1^ with AITC.

The steady-state kinetic parameters for the reaction with Δ^5^-androsten-3,17-dione were determined for the two enzymes (Table 5).

**Table 5:** Kinetic parameters for the ketosteroid isomerization of Δ^5^-AD.

Enzyme	K_m_ (mM)	k_cat_ (s^-1^)	k_cat_/K_m_ (mM^-1^ s^-1^)
Marmoset GST A3-3	0.374 ± 0.087	261 ± 49	698
Marmoset GST A1-1	0.303 ± 0.034	6.89 ± 0.79	22.7
Human GST A3-3	0.024 ± 0.004	204 ± 22	8600 ± 800
Equine GST A3-3	0.0137 ± 0.0015	219.2 ± 7.9	16000 ± 1900

**Fig. 5:**
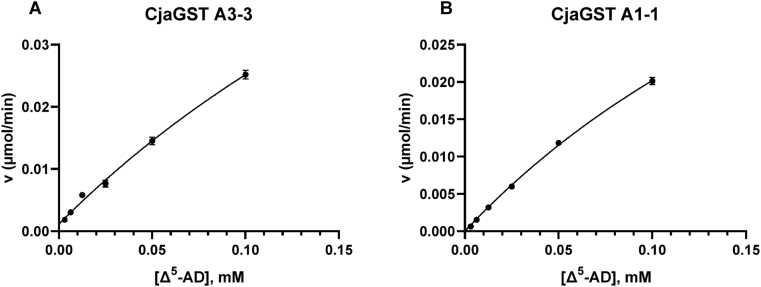
Dependence of initial rates of double-bond isomerization for the two marmoset GSTs. Measurements were made in triplicate in the standard assay system with different concentrations of Δ^5^-AD and a fixed concentration of 1.0 mM glutathione.

The dependence of the initial velocity on the Δ^5^-AD concentration in the range 0.005–0.10 mM was determined with the two marmoset GSTs. Saturation was not reached due to the limited solubility of the substrate (Fig. 5A and B). Measurements were made in triplicate at 30 °C and pH 8.0 in the standard reaction system. The Michaelis-Menten equation was fitted by nonlinear regression. For comparison the parameters previously determined for the human and equine orthologs are shown [4] (Table 5).

### 3.4 Inhibition studies

In order to further probe the similarities of CjaGST A3-3 with its orthologs, the effects on the enzyme of two inhibitors previously studied were investigated.

**Fig. 6:**
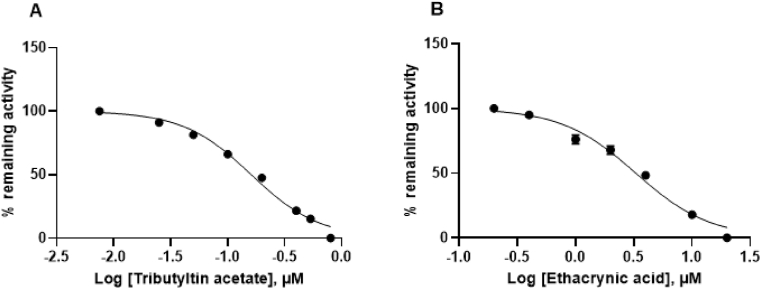
Inhibition of CjaGST A3-3 by the organotin compound tributyltin acetate (A) and the diuretic drug ethacrynic acid (B). Remaining enzyme activity measured with CDNB as substrate in the standard assay system was determined at different inhibitor concentrations. The measurements were carried out in triplicate and the data were subjected to nonlinear regression analysis.

Tributyltin acetate was a potent inhibitor with an IC_50_ value of 0.16 ± 0.004 μM (Fig. 6A). The same compound gave IC_50_ values of 0.28 ± 0.02 μM for EcaGST A3-3 [16] and 0.0023 μM for HsaGST A3-3 [2]. Ethacrynic acid likewise inhibited CjaGST A3-3 with an IC_50_ value of 3.3 ± 0.2 μM (Fig. 6B). For comparison IC_50_ values for ethacrynic acid of 0.18 ± 0.067 μM for EcaGST A3-3 [17] and 0.4 μM for HsaGST A3-3 [18]. Thus, CjaGST A3-3 is strongly inhibited by the same inhibitors as the equine and human ortholog, even if the IC_50_ values differ considerably.

## 4 Discussion

The present investigation demonstrates that the common marmoset *Callithrix jacchus* encodes GST enzymes with steroid isomerase activity. The most active enzyme is CjaGST A3-3 showing a specific activity with Δ^5^-AD of 62.1 ± 1.8 μmol min^-1^ mg^-1^, approximately 3-fold lower than the activities of HsaGST A3-3 and EcaGST A3-3. On the other hand, the marmoset enzyme matches porcine SscGST A2-2, which is most active ketosteroid isomerase known in the pig with a specific activity of 53 μmol min^-1^ mg^-1^ [5]. It would thus appear that CjaGST A3-3 can contribute to steroid hormone biosynthesis, as demonstrated for the human ortholog HsaGST A3-3 [2].

The steady-state kinetics show that the k_cat_ values of the three GST A3-3 enzymes, marmoset, human, and equine, are all approximately 200 s^-1^ (Table 5), but the k_cat_/K_m_ values span a range from 698 to 16,000 mM^-1^ s^-1^ with CjaGST A3-3 showing the lowest catalytic efficiency. The differences in efficiencies among the enzymes arise mainly from the K_m_ values, which range between 0.374 and 0.0137 mM. The marmoset catalytic efficiency of 698 mM^-1^ s^-1^ is similar to the porcine SscGST A2-2 of 800 mM^-1^ s^-1^ [5].

In comparison with CjaGST A3-3, CjaGST A1-1 has a 30-fold lower specific activity with Δ^5^-AD (Table 4) and a 37-fold lower k_cat_ value. The catalytic efficiency of CjaGST A1-1 is 31-fold lower than the value of CjaGST A3-3.

Two residues, 111 and 213, differ in the H-sites of the 91.4% sequence identical CjaGST A1-1 and CjaGST A3-3, and the modeling of the structures suggests that the bulky phenyl ring of Phe111 in CjaGST A1-1 causes steric hindrance in the binding of the steroid substrate Δ^5^-AD (Fig. 4).The second residue Leu213 in CjaGST A1-1 is somewhat larger than Val213 in CjaGST A3-3 and may thus further limit the accessible space for substrate binding. On the other hand, the highly efficient HsaGST A3-3 also has Leu213 in the corresponding position, showing that this residue is not incompatible with high ketosteroid isomerase activity.

Comparison of CjaGST A3-3 with the more efficient homologous human and equine enzymes suggests that the higher ketosteroid isomerase activity is due to the smaller residues Gly12 and Ala/Gly208 than the residues Ala12 and Thr208 in CjaGST A3-3. However, it is well established that catalytic efficiency of an enzyme involves the collective action of many parts of the protein and is governed by contributions not limited by the active-site architecture, such that the influence of individual amino acid residues is difficult to evaluate.

Recently, 20 marmoset GSTs were cloned and characterized, with a total of four Alpha class enzymes including CjaGST A1-1 and CjaGST A3-3 encoded on chromosome 4 [19]. With reference to our prior work on human GSTs [1,20,21] Uno et al. proposed that CjaGST A1-1 in the marmoset GSTs would have steroid isomerase activity, but no experimental support was presented. Our current studies show that a marmoset enzyme indeed displays high ketosteroid isomerase activity with both Δ^5^-AD and Δ^5^-PD, but that the main activity resides with CjaGST A3-3. Surprisingly, no indication of high expression of CjaGST A3-3 in the typical steroidogenic organs were found in the marmoset [19]. However, not tested were placenta, ovary, and adrenal gland where the highest levels have been noted in human [1] and equine [4] tissues. Further investigations are required to clarify how the marmoset enzyme can contribute its potent steroid isomerase activity to physiology.

## Author contributions

**Bengt Mannervik:** Conceptualization, Writing—original draft preparation, Writing—reviewing and editing, Supervision, Project administration, Funding acquisition; **Aram Ismail:** Writing—reviewing and editing, Formal analysis, Visualization, Investigation; **Julia Sawmi:** Writing—reviewing and editing, Formal analysis, Visualization, Investigation.

## Declaration of competing interest

The authors declare no conflicts of interest.

## References

[1] Johansson A.S., Mannervik B. (2001). Human glutathione transferase A3-3, a highly efficient catalyst of double-bond isomerization in the biosynthetic pathway of steroid hormones. J. Biol. Chem..

[2] Raffalli-Mathieu F., Orre C., Stridsberg M., Hansson Edalat M., Mannervik B. (2008). Targeting human glutathione transferase A3-3 attenuates progesterone production in human steroidogenic cells. Biochem. J..

[3] Benson A.M., Talalay P. (1976). Role of reduced glutathione in the delta(5)-3-kitosteroid isomerase reaction of liver. Biochem. Biophys. Res. Commun..

[4] Lindström H., Peer S.M., Ing N.H., Mannervik B. (2018). Characterization of equine GST A3-3 as a steroid isomerase. J. Steroid Biochem. Mol. Biol..

[5] Fedulova N., Raffalli-Mathieu F., Mannervik B. (2010). Porcine glutathione transferase Alpha 2-2 is a human GST A3-3 analogue that catalyses steroid double-bond isomerization. Biochem. J..

[6] Worley K.C., Warren W.C., Rogers J., Locke D., Muzny D.M. (2014). The common marmoset genome provides insight into primate biology and evolution. Nat. Genet..

[7] Nese H.A., Mannervik B., Raffalli-Mathieu F. (2012). Molecular cloning, expression, purification and characterization of wild type and mutant marmoset alpha class glutathione transferase. FEBS J..

[8] Porath J., Carlsson J., Olsson I., Belfrage G. (1975). Metal chelate affinity chromatography, a new approach to protein fractionation. Nature.

[9] Pettersen E.F., Goddard T.D., Huang C.C., Couch G.S., Greenblatt D.M., Meng E.C., Ferrin T.E. (2004). UCSF Chimera--a visualization system for exploratory research and analysis. J. Comput. Chem..

[10] Sali A., Blundell T.L. (1993). Comparative protein modelling by satisfaction of spatial restraints. J. Mol. Biol..

[11] Mannervik B., Board P.G., Hayes J.D., Listowsky I., Pearson W.R. (2005). Nomenclature for mammalian soluble glutathione transferases. Methods Enzymol..

[12] Mannervik B., Guthenberg C., Jakobson I., Warholm M., Aitio A. (1978). Glutathione conjugation: reaction mechanism of glutathione S-transferase A. Conjugation Reactions in Drug Biotransformation..

[13] Sinning I., Kleywegt G.J., Cowan S.W., Reinemer P., Dirr H.W., Huber R., Gilliland G.L., Armstrong R.N., Ji X., Board P.G. (1993). Structure determination and refinement of human alpha class glutathione transferase A1-1, and a comparison with the Mu and Pi class enzymes. J. Mol. Biol..

[14] Tars K., Olin B., Mannervik B. (2010). Structural basis for featuring of steroid isomerase activity in alpha class glutathione transferases. J. Mol. Biol..

[15] Škerlová J., Lindström H., Gonis E., Sjödin B., Neiers F., Stenmark P., Mannervik B. (2020). Structure and steroid isomerase activity of Drosophila glutathione transferase E14 essential for ecdysteroid biosynthesis. FEBS Lett..

[16] Škerlová J., Ismail A., Lindström H., Sjödin B., Mannervik B., Stenmark P. (2021). Structural and functional analysis of the inhibition of equine glutathione transferase A3-3 by organotin endocrine disrupting pollutants. Environ. Pollut..

[17] Lindström H., Mazari A.M.A., Musdal Y., Mannervik B. (2019). Potent inhibitors of equine steroid isomerase EcaGST A3-3. PloS One.

[18] Musdal Y., Hegazy U.M., Aksoy Y., Mannervik B. (2013). FDA-approved drugs and other compounds tested as inhibitors of human glutathione transferase P1-1. Chem. Biol. Interact..

[19] Uno Y., Uehara S., Tanaka S., Murayama N., Yamazaki H. (2020). Systematic characterization of glutathione S-transferases in common marmosets. Biochem. Pharmacol..

[20] Johansson A.S., Mannervik B. (2002). Active-site residues governing high steroid isomerase activity in human glutathione transferase A3-3. J. Biol. Chem..

[21] Pettersson P.L., Mannervik B. (2001). The role of glutathione in the isomerization of delta 5-androstene-3,17-dione catalyzed by human glutathione transferase A1-1. J. Biol. Chem..

